# Miscellaneous

**Published:** 2008-10

**645**

**Evaluation of antihypercholesteremic activity of indigenous plants using male sprague-dawley rats**

Vithlani D, Sharma V, Sathaye SS

**University Institute of Chemical Technology, Mumbai, India.**

**Introduction and Objective:**

Cardiovascular diseases continue to be a leading cause of morbidity and mortality in developed as well as developing countries. Hypercholesteremia is one of the factors that have contributed in acceleration of Coronary Heart Diseases in India. There is a necessity to prove the efficacy of plant based drugs scientifically and systematically. To evaluate the antihypercholesteremic activity of individual methanolic extract as well as combination (1:1:1) of methanolic extract of Trigonella foenumm, Trachyspermum copticum and Nigella sativa at two different doses using male Sprague-Dawley rats.

**Method:**

The Sprague Dawley rats were administered Cholesterol (1 gm/kg) along with Cholic acid (125 mg/kg) dispersed in groundnut oil for 6 weeks to all groups containing 6 animals per group except Control group. Blood samples were collected on day 0 (baseline values), day 14, day 28, day 42 and biochemical parameters like Cholesterol, Triglyceride, HDL, LDL, VLDL, Atherogenic index were estimated. Then groups were treated with methanolic extracts of the three plants as well as the combination of all the three extracts in 1:1:1 ratio at two different dose levels (100 mg/ml and 200 mg/ml) for 14 days. Blood was collected on day 56 and same parameters were estimated, to evaluate antihyperlipidemic activity of the same.

**Result and conclusions:**

The combination of methanolic extract of all the three plants in equal proportion showed better antihypercholesteremic activity in dose dependent manner than methanolic extract of individual plant in Sprague-Dawley male rats. All the methanolic extracts showed significant increase in excretion of fecal cholesterol. The activity of the enzyme was significantly depressed by Atorvastatin calcium as well as all methanolic extracts except *T. copticum* (Low dose).

**646**

**Profile of 52 cases of leukemia: A retrospective analysis with reference to immunophenotyping**

Bhatt JD^1^, Ghatak SB^1^, Patel AP^2^, Patel JA^1^

**^1^Institute of Pharmacy, Nirma University of Science & Technology, Ahmedabad, India; ^2^Hematology Clinic, Navrangpura, Ahmedabad, India.**

**Introduction:**

Leukemia is malignant neoplasm of hematopoetic cells originating from bone marrow. Its diagnosis and treatment depend on unequivocal detection and identification of hematopoetic cell population and lineage. The present study was designed to analyze the impact of immunophenotyping in early detection and management of leukemias.

**Method:**

Retrospective analysis on 52 patients (36 males, 16 females) attending Hematologic Clinic, Ahmedabad was conducted from January 1999 to February 2008 with special emphasis on immunophenotyping along with epidemiology, cytochemistry and cytogenetics in diagnosis and management of leukemia.

**Results:**

Immunophenotyping revealed 91.18% of B-acute lymphoid leukemia cases, 8.82% of T- acute lymphoid leukemia cases, 12.5% of acute myeloid leukemia cases, 12.5% each of chronic lymphoid leukemia cases (stage 1 and stage 2), 7.5% of chronic lymphoid leukemia cases (stage 0) and 1.92% each of hairy cell leukemia and NK-cell leukemia. The most significant distinctive immunological markers between acute myeloid leukemia (M0) and acute lymphoid leukemia were found to be CD33 and myeloperoxidase. Reciprocal translocation and hyperploidy (38.46%) were the most common chromosomal abnormalities present. Myeloperoxidase test was positive in 2 (50%) patients of acute myeloid leukemia. Specific chemotherapy regimes were administered to 47 (90.38%) patients differing according to type of leukemia. 82.7% patients were alive and 17.3% patients were dead at the time of last follow up.

**Conclusions:**

Major diagnostic role of immunophenotyping is to differentiate between immature acute myeloid leukemias (M0) and acute lymphoid leukemias. Immunophenotyping studies along with cytochemistry and cytogenetics are essential for early detection and faster eradication of acute leukemias.

**647**

**Drug prescribing pattern in a tertiary care teaching institution in north India**

Kumar R, Ram J, Singh B, Kaur C

**Guru Gobind Singh Medical College (BFUHS), FARIDKOT, PUNJAB- India.**

**Objective:**

To study drug prescribing pattern in the Tertiary Care teaching Hospital in North India.

**Materials and Methods:**

Prescriptions were randomly collected from the OPD over a period of three months and relevant information was entered into pretested Performa and analyzed for parameters.

**Results:**

Total number of prescription: 100. Total number of drugs prescribed: 468. Average number of drugs per prescription: 4.68. The percentage of drugs from essential medicine list (EML) was 18% while percentage of drugs prescribed by generic name was nil. Most common groups of drugs prescribed by physicians were antibiotics 11.32% (53), NSAIDs 7.7% (36), multivitamins 4.48% (21), antacids 3.85% (18), antihistaminics 2.56% (12), and 1.71% (8) corticosteroids. Drugs prescribed in Injection forms were 14.53% (68) and illegible prescriptions were 24% that contain 32.43% of drugs out of total.

**Conclusion:**

For rational prescribing of drugs there is a need of mass awareness amongst physicians about the good prescribing by following WHO program on rational use of drugs.

**648**

**Disposition kinetics of sparfloxacin in goat after intravenous administration in induced acute renal failure goats**

Karmakar UK, Jha KC, Datta BK, Chatterjee US, Sar TK, Chakraborty AK, Mandal TK

**West Bengal University of Animal and Fishery Sciences, Kolkata, India.**

**Objective:**

Sparfloxacin is a potent, long acting third generation bactericidal fluoroquinolone derivative acute renal failure may alter kinetic behavior of Sparfloxacin. Present study was undertaken to establish the dosage regimen of sparfloxacin in healthy as well as acute renal failure condition of goat.

**Materials and Methods:**

Eight goats of either sex were divided into two groups. Group I goats served as control healthy goats where as Group II was induced acute renal failure by blocking of urinary bladder using Foley’s Catheter. Sparfloxacin was administered intravenously at the dose rate of 30 mg kg-1 body weight to both groups. Blood samples were collected at different time interval to study disposition kinetics. Plasma protein binding activity and kidney function test were also performed in this study.

**Result and Discussion:**

Mean value of BUN and CRT in plasma of group II goats were significantly increased at 60 hr. Sparfloxacin were detected in group II goats up to 14 hr where as 10 hr in group I goats. Concentration of sparfloxacin was higher in group II goats. Pharmacokinetic parameter like β, Vd_area_, ClB were higher in healthy goats where as β, t½β, AUC, MRT were higher in group II goats. The percentage of plasma protein binding activity of Sparfloxacin in group II goats was non significantly higher than healthy goats.

**Conclusion:**

Pharmacokinetic profiles of Sparfloxacin were altered in acute renal failure condition of goats.

**649**

**Evaluation of analgesic and anti-inflammatory activity of bark extract of *Neolamarckia cadamba* in rodents**

Yadav CH^1^, Jain SK^1^, Wanjari MM^2^

**^1^Institute of Pharmacy, Bundelkhand University, Jhansi, India; ^2^Central Research Institute (Ayurveda), Gwalior, India.**

**Introduction:**

The stem bark of Neolamarckia Cadamba (Rubiaceae) is used in traditional system of medicine for treatment of pain and inflammatory conditions. Therefore, it was proposed to evaluate analgesic and anti-inflammatory activity of methanolic extract of Neolamarckia cadamba bark in rodents.

**Methods:**

The methanolic extract of Neolamarckia cadamba bark (NCBE) was subjected to phytochemical screening and acute toxicity study in rats. The analgesic effect was studied using acetic acid-induced writhing test and hot plate analgesia model in mice. The anti-inflammatory effect was investigated in rats using Carrageenan-induced paw oedema (Acute inflammation) and cotton pellet granuloma model (Chronic inflammation). NCBE was administered in graded doses 100, 200,400 and 800 mg/kg per orally (p.o.) singly or up to seven days. Morphine (100mg/kg), aspirin (100mg/kg) and indomethacin (10mg/kg) p.o. were used as standard drugs.

**Results:**

NCBE was found to be safe up to 2000 mg/kg, p.o. (LD50>2000mg/kg). NCBE (100-800 mg/kg p.o) showed significant dose dependent decrease in the number of acetic acid-induced writhing movements and increase in the reaction time in hot plate analgesia test. NCBE also showed significant dose dependent inhibition of carrageenan induced paw oedema and formation of cotton pellet granuloma tissue.

**Conclusion:**

Investigations revealed that NCBE exhibits analgesic and anti-inflammatory effect possibly through inhibition of inflammatory mediators and prostaglandin synthesis. These effects of NCBE may be due to the presence of alkaloids, triterpenoids, sterols and flavonoids.

**650**

**Anti-oxidant activity of dried seeds of *Trigonella foenumm* (fenugreek), *Nigella sativa* (black cumin) and *Linum lusitatissimum* (linseed) and dried ripe fruits of *Trachyspermum copticum* (ajowan)**

Kenjale R Vithlani D, Sharma V, Shaikh MF, Shah D, Shete H, Sathaye S

**Institute of Chemical Technology, Mumbai, India.**

Objective of present study was to evaluate anti-oxidant potential of methanolic extracts of dried seeds of Trigonella foenumm (TF), Nigella sativa (NS) and dried ripe fruits of Trachyspermum copticum (TC) individually and as combined extract (CE) in ratio 1:1:1 respectively and of water-methanolic (1:1) extract of Linseed seeds. Anti-oxidant activity evaluation was done using Folin Ciocalteu method for total phenolic content, Oyaizu Method for total reduction capacity, DPPH free radical scavenging assay and TBARS assay for lipid peroxidation activity. Results showed that TC possesses highest phenolic content (464.239 ± 10.04 mg equivalent of Gallic acid/gm of extract) followed by that of CE (322.87 ± 6.31), Linseed, NS and TF. All four extracts showed electron donating capacity in a concentration dependent manner. Methanolic extract of TC (1.596 ± 0.16) was the most potent reducing agent followed by that of Linseed (1.146 ± 0.031), CE, NS and TF However, the capacities were inferior to that of ascorbic acid and gallic acid. CE (IC50 value: 107.85*µ*g/ml) was found to be most active free radical scavenger followed by TC (316.19*µ*g/ml), Linseed (426.11*µ*g/ml), NS (457.95*µ*g/ml) and TF. However, the methanolic extracts were not as effective as Ascorbic acid (61.67*µ*g/ml). All four extracts prevented formation of TBARS in a concentration dependent manner. Linseed was found to be most active followed by CE, TC, NS and TF respectively. Thus, methanolic extract of dried ripe fruits of Trachyspermum copticum, water-methanolic extract of Linseed and combined methanolic extracts of TC, NS and TF have shown promising anti-oxidant potential.

**651**

**Antivenom activity of a dialdehyde compound from*Curcuma*sp. Rhizome against *Naja kaouthia* venom**

Sattayasai J^1^, Phimmasone S^1^, Puapairoj P^1^, Sattayasai N^2^, Lattmann E^3^

**^1^Department of Pharmacology, Faculty of Medicine; ^2^Department of Biochemistry, Faculty of Science, Khon Kaen University, Khon Kaen, Thailand; ^3^Aston Pharmacy School, Aston University, Birmingham, England.**

Many plants of Curcuma sp. (Zingiberaceae) are reputed in Thailand as “snakebite antidote”. The rhizomes of these plants were found to have a common yellowish substance. The substance was shown to be a dialdehyde compound and believed to be an active substance. In this study, a dialdehyde compound isolated from Wan-Khor-Thong, a Curcuma sp., was tested both in vitro and in vivo for antivenom activity against Naja kaouthia (cobra) venom. The compound (in 95% ethanol) showed a significant (*P* < 0.01) antagonistic effect on the inhibition of neuromuscular transmission produced by cobra venom in isolated rat phrenic nerve-hemidiaphragm preparations. Mice, subcutaneously (SC) injected with venom at 0.75 mg/kg, had the survival time of 52.00 ± 5.35 min, while giving dialdehyde compound at a dose of 100 mg/kg (in 5% DMSO) orally or intraperitoneally, 30 min before the venom, had survival time of 61.50 ± 5.38 and 88.75 ± 11.15 min, respectively. Mice received the mixture (SC) of venom (0.75 mg/kg) and the compound (100 mg/kg), previously incubated at 37°C for 30 min, were all survive. Injection (SC) of dialdehyde compound, immediately after the venom, also significantly increased animal survival time. The present finding suggests that a dialdehyde compound from Curcuma sp. rhizome has the antagonistic effect against the cobra venom.

**652**

**Kajjali, an ayurvedic herbomineral preparation, accelerates the gastric emptying in experimental sprague dawley rats**

Shete H K, Shah D P, Shaikh M F, Sathaye S S

**University Institute of Chemical Technology, Mumbai, India.**

**Introduction:**

Kajjali an Ayurvedic herbomineral preparation, prepared from mercury and sulphur as per ayurvedic texts, has been reported to have a bioavailability enhancement effect on the compounds co-administered with it. Bioavailability of compounds, which are acid labile or whose absorption is primarily from intestine, can be enhanced by its rapid gastric emptying.

**Objective:**

The objective of the present work was to study the effect of kajjali on gastric emptying of phenol red meal.

**Materials and Methods:**

An UV-visible spectrophotometric method (λmax= 560nm) was developed for the estimation of phenol red content in the stomach matrix. Healthy male Sprague dawley (230-250g) rats were divided into four groups. (N=8 per group). The animals from group-I received kajjali (12mg/kg p.o) for 15 days and the group-II received vehicle. Group-III received the standard drug i.e. metoclopramide (3mg/kg p.o) whereas the group-IV received no treatment at all, served as a control. All rats were given test meal of methyl cellulose mixed with phenol red on the final day of the study. The effect on the test meal emptying was studied by measuring the phenol red content in the stomach after 20 mins of meal administration.

**Results and Conclusion:**

Both kajjali and metoclopramide significantly increased the gastric emptying of the phenol red (*P* < 0.01). Present study indicates the role of kajjali in gastric emptying which could be possible reason for its bioavailability enhancing effect for the acid labile drugs and for the drugs whose primary site of absorption is intestine.

**653**

**Study of metformin against experimental gastric ulcers**

Savaliya JT, Jain SM

**L. M. College of Pharmacy, Ahmedabad, India.**

**Introduction:**

In the present study, metformin, a biguanide derivative of antidiabetic was studied for its anti-ulcer and antioxidant effects in gastro-intestinal disorder.

**Methods:**

An antiulcer effect of metformin was investigated against ethanol-induced gastric mucosal damage, pylorus ligation and water immersion plus restrain stress-induced gastric ulcers in rats. Ulcer index was a common evaluating parameter in all the models. In the pylorus ligation model, acid secretory parameters (pepsin activity, total acidity and total acid output) and mucoprotective parameters (total carbohydrate, total protein and mucin activity) were studied. In addition, lipid peroxidation and antioxidant activities (super oxide dismutase, catalase and nitric oxide) were specifically studied in water immersion plus restraint stress-induced gastric ulcer model.

**Results:**

Metformin (200mg/kg, p.o.) showed significant protection against gastric ulceration as evident from reduction (*P*<0.05) in ulcer index in all the models and results were comparable with that of Omeprazole (20mg/kg, p.o.), a reference standard. In the pylorus ligation model, metformin showed significant increase in mucin activity and decrease (*P*<0.05) in acid secretory parameters. Besides, metformin pretreatment showed reduction in malondialdehyde content and myeloparoxidase levels.

**Conclusions:**

Hence, the results of our study provide some new information that metformin possesses significant anti-ulcer activity. The mechanism of its activity can be attributed to decrease in acid secretion and lipid paroxidation alongwith cytoprotection and thereby strengthening of gastric mucosal barrier.

**654**

**Effects of combinations of *Tinospora cordifolia* (TC) and *Phyllanthus emblica* (PE) with and without *Ocimum sanctum* (OS) on B lymphocyte activity: An experimental study**

Shetty V, Nigade J, More D, Sawant S, Uchil D, Kamat SK, Rege NN

**Seth GS Medical College and KEM Hospital, Parel, Mumbai - 400 012, India.**

**Introduction:**

The present study was undertaken to evaluate combination of Tc and Pe (1:3) with or without Os coating on B lymphocytes.

**Methods:**

Following Animal Ethics Committee approval, Swiss albino mice (20-30 gms, either sex) were divided into 3 groups, Group A, B and C. In each group mice were further divided into; Gp1: vehicle, Gp2: Tc (100 mg/kg/d), Gp3 : Tc+Pe (400mg/kg/d) and Gp 4: Tc+Pe+Os (400mg/kg/d). In Gp A, on 16th day of therapy, thymus dependent antigen, KLH was injected (100 *µ*g i.p). Treatment was continued till d30. IgM and IgG were assessed on d15, 23 and 30 using ELISA. In Gp B, on 16th day, splenic proliferation in response to 0.025 microg/ml LPS, a thymus independent antigen was studied using tritiated thymidine. In Gp C i.e. treated hemisplenectomized mice, antibody response to KLH was studied.

**Results:**

In normal mice, all plant drugs increased IgM and IgG titer compared to Gp1. Maximum response was with Tc+Pe+Os; Tc+Pe was comparable to Tc. Splenocytes induced by Tc and both the combinations also showed increased thymidine uptake but difference amongst the groups was not significant. In hemisplenectomized mice, IgM and IgG titer was higher in drug treated groups than in Gp 1; the order being Tc+Pe+Os > Tc+Pe> Tc.

**Conclusion:**

Tc+Pe combinations activate B lymphocytes in normal and hemisplenectomised mice. However, splenocyte proliferation induced by them was comparable to Tc alone. Maximum beneficial effect was offered by Tc+Pe coated with Os, a process recommended in Ayurveda to potentiate drug effects.

**655**

**Comparative study of efficacy and safety of ketarolac tromethamine 0.4% And prednisolone acetate 1% in controlling postoperative inflammation after small incision cataract surgery**

Sathish Kumar M, Venkateswaran J, Christina AJM, Azeez SA, Sailaja G

**KM College of Pharmacy, Madurai, Tamilnadu,Aravind Eye Hospitals, Madurai, Tamilnadu, India.**

Cataract is clouding of lens in the eyes that affects vision. Most cataracts are related to aging. Small incision cataract surgery is one of the five surgical techniques to remove cataract. After this surgery there may be some postoperative complications such as wound leak, endophthalmitis, corneal edema, glaucoma etc… A topical steroid such as prednisolone acetate and ketarolac tromethamine has been used in combination with topical antibiotics to control infection and inflammation. The objective of this study is to compare the efficacy and safety of topical NSAID and corticosteroid in controlling postoperative inflammation after small incision cataract surgery. This double-blind, randomized, single-site study comprised 57 patients with clinical diagnosis of routine ocular cataract requiring surgical removal. All patients had small incision cataract surgery. After surgery, patients were randomized to receive ketarolac tromethamine 0.4% or prednisolone acetate 1% for a period of six weeks. The intra ocular anti-inflammatory drug efficacy was assessed by lid edema, ciliary flush, corneal edema, anterior chamber reaction, intraocular pressure, fibrinous uveitis,/ hypopyon, cystoid macular edema, visual acuity with snellen’s chart and slit lamp examination postoperatively on days 1, 7, 30 and 3 rd month. In the current study we found that there were no intraoperative complications during surgery. Postoperatively, lid edema, ciliary flush, corneal edema, anterior chamber reaction, intraocular pressure and visual acuity there was no significant difference between the two treatment groups. Fibrinous uveitis, / hypopyon, cystoid macular edema was absent in all the follow up of both treatment groups. This trial shows that both ketarolac tromethamine 0.4% or prednisolone acetate 1% ophthalmic solutions are equally effective and safe in controlling postoperative inflammation in patients undergoing small incision cataract surgery.

**656**

**Evaluation of anti-Inflammatory activity of ethanolic extract of *Acalypha indica* Linn.**

Sandip K, Manjunath PM, Anitha S, Divakar Goli, Uday Raj Sharma

**Acharya and B.M Reddy College of Pharmacy, Bangalore, India.**

Acalypha indica was collected from the region of Hessaraghatta and was authenticated by the Department of Botany, Gnanabharathi, Bangalore. The fresh leaf of Acalypha indica was air dried and ground into fine powder. The finely powdered leaf was extracted with ethanol. The ethanolic extract was used for the evaluation of anti-inflammatory activity. 1% yeast suspension was used to induce inflammation in rat paw. The subcutaneous injection of 1% yeast induced inflammation in rats. The inflammation produced was measured plethysmometrically at the time intervals of 0, 1, 3, and 24 hours. The ethanolic extract was administered orally for the evaluation off anti-inflammatory activity. The significant (p<0.05) activity was observed at the dose of 200mg/kg body weight after 24 hours. The percentage inhibition of paw oedema at 200mg/kg body weight was found to be 71.42%.

**657**

**Effect of adenosine on pulmonary angiogenesis and respiratory functions in egg albumin sensitized rats**

Patel LH^1^, Shah GB^2^

**^1^Ramanbhai Patel College of Pharmacy, Changa, India; ^2^K.B.Institute of Pharmaceutical Education and Research, Ghandhinagar, India.**

**Introduction and Objective:**

Elevated levels of adenosine have been observed in bronchoalveolar fluid of patients with asthma. A high level of adenosine in pathologic conditions is reported to act through A2B receptors, which are otherwise less functional. Adenosine via A2B is therefore likely to be involved in mediating angiogenesis, inflammation and hyper responsive status in asthma. In this context, the present study was aimed to confirm whether adenosine plays any role in pathogenesis of asthma and if yes whether A2B receptors are involved in this activity.

**Materials and Methods:**

For this adenosine (200ug/ ml) was administered by aerosol to both egg albumin sensitized and unsensitized rats. The respiratory functions and arterial density in the lungs of these animals ware assessed. Additionally lithium carbonate was administered to group of sensitized animals along with adenosine to see whether A2B receptor activation and consequent Gq protein is involved in adenosine action.

**Result:**

As compared to unsensitized animals, adenosine administration to sensitized animals resulted in more arterial density (angiogenesis), higher serum bicarbonate level, lower serum pO2 and low air flow rate was observed. In animals who received additional lithium carbonate treatment, these adverse changes in respiratory functions were not observed.

**Conclusion:**

Studies revealed that, adenosine induces angiogenesis and adverse respiratory function changes in asthmatic conditions. These effects are probably mediated though A2B receptors. However, more detail and direct studies using selective A2B receptor ligands could give final confirmation.

**658**

**Evaluation of the effect of CYP450 modulators in diabetic cataractogenic rats**

Shah A, Patel S, Patel K, Gandhi T

**Anand Pharmacy College, Anand, India.**

**Objective:**

To evaluate the effect of CYP450 modulators in diabetic cataractogenic rats.

**Method:**

Male SD suckling rats (18 days; 40-50 gms.) were randomly allocated to four groups (n=15). Group I animals were fed with normal laboratory chow whereas the animals of Group II, III and IV were fed with galactose (50 %) diet for 18 days starting from day 21 after parturition. Three days prior to the galactose feeding treatment with Pioglitazone (3.8 mg/kg; P.O; once daily) and Verapamil (40 mg/kg; P.O; once daily) was administered to Group III and IV respectively and continued till the end of study. Gross examination (daily) and Microscopical examination (every 3rd day) was done. Three animals from each group were sacrificed on 4th, 9th, and 12th day of galactose feeding and the remaining animals were sacrificed on 18th day to estimate various biochemical parameters.

**Result:**

Gross examination, microscopical examination and SEM photographs revealed that Pioglitazone treatment induced whereas Verapamil treatment delayed the appearance of cataract as compared to model control. Pioglitazone treatment decreased soluble & total protein, GSH, -SH and increased AR, insoluble protein, intracellular calcium, and MDA on day 4, 9, 12, 18 corresponding to stages (I-IV) of cataract in model control. Verapamil treatment increased soluble & total protein GSH,-SH and decreased AR, insoluble protein, intracellular calcium on day 4, 9, 12, 18 corresponding to stages (I-IV) of cataract in model control.

**Conclusion:**

Pioglitazone treatment induces the cataract progression whereas Verapamil treatment delayed the cataract development.

**659**

**Anticonvulsant and neurotoxicity profile of *Lavandula stoechas* L. Flower essential oil**

Zaidi SMA^1^, Pathan SA^1^, Ahmad FJ^1^, S Surender^2^, Vohora D^1^, Iqbal Z^1^, Talegaonkar S^1^, Jamil S^1^, Khar RK^1^

**^1^Jamia Hamdard University, New Delhi, ^2^All India Institute of Medical Sciences, New Delhi, India.**

**Introduction:**

***Lavandula stoechas* L**., (Ustukhudus) Fam. Lamiaceae has been used in the Unani system of medicine from ancient times to treat various brain disorders especially epilepsy. The aqueous and alcoholic extract of Lavandula stoechas L. flowers have already been reported for its various CNS related activities. In the current study, essential oil obtained from Lavandula stoechas L. flowers by hydrodistillation was evaluated for its anticonvulsant activity and neurotoxicity.

**Materials and Methods:**

Lavandula essential oil at a dose range from 0.3mL/kg to 1.2 mL/kg (p.o.) screened for anticonvulsant activity with Pentylenetetrazole (PTZ, 70mg/kg, i.p.), Increased current electroshock (ICES) models, while rotarod test was employed for neurotoxicity in Swiss Albino mice.

**Results:**

The essential oil increased the latency period for myoclonic jerks and generalized clonic seizures for PTZ at highly significant level in a dose dependent manner at a maximum dose of 0.9 mL/kg. The results demonstrated a significant increase in the seizure threshold current for ICES in a non dose dependent manner. Essential oil had no effect on motor activity upto a dose of 0.9mL/kg at all time intervals, however higher doses elicited motor impairment at all time intervals on rotarod. Acute toxicity study for the essential oil was also conducted and LD50 estimated at 2.5 mL/kg.

**Conclusion:**

The Lavandula essential oil possesses anticonvulsant activity and could be attributed to the presence of variety of terpenes in the essential oil.

**660**

**Neuroprotective effect of dopamine receptor antagonist (pimozide) in diabetes induced hyperalgesia in rats**

Shah PH^1^,Patel NR^2^, Kanzariya NR^1^, Patel N^1^, Patel Rameshvar K^1^, Patel NJ^1^

**^1^S.K. Patel College of Pharmaceutical Education & Research, Mehsana, ^2^Kalol Institute of pharmacy, Kalol, India.**

**Objective:**

Neuropathy results from diabetic microvascular injury to small blood vessels that supply blood to nerves. Neuropathic pain in diabetics is characterized by increase in pain sensation, which is known as hyperalgesia. For the development of hyperalgesic condition in diabetes various mediators and pathways are proposed. In the present study we are trying to decipher role of dopaminergic pathway in diabetes induced hyperalgesia.

**Methods:**

Diabetes was 2induced in male wistar rats by administration of streptozotocine i.p (60mg/kg). The screening models used for evaluation of hyperalgesia were radial heat tail flick and Eddy’s hot plate method, which aid in elucidation of supraspinally nociceptive integrated responses.

**Results:**

The STZ challenge was capable of triggering a hyperalgesic response, which took 2 weeks for onset and remains significant for at least 6 weeks. The amplitude of hyperalgesia in diabetic rats was significantly reduced in pimozide treated rats as compared to disease control group. In both methods time latency was increased significantly at 6 weeks in treated group. Moreover, contractile response of dopamine in rat anococcygeus muscle help in elucidation of the dopaminergic system activation which was infered by significant increase in pD2 value of dopamine in diabetic rats as compared to normal rats. Thus the dopaminergic receptor antagonistic effect of pimozide was thought to be involved in reducing the pain response.

**Conclusion:**

From the results, we infer that dopaminergic system activation in the diabetic condition follows the early induction of hyperalgesia and Pimozide decreases diabetes induced hyperalgesia by antagonizing action of dopamine receptors

**661**

**Evaluation of antihyperlipidimic activity of saponin fraction of roots of *Momordica cymbalaria***

Sandeep S, Koneri R, Mushirbhai M I

**Visveswarapura Institute of Pharmaceutical Sciences, Bangalore, India.**

**Introduction:**

Atherosclerosis is the leading cause of death in the developed and developing countries like India. It is associated with elevated lipid levels in the blood. Treatment of hyperlipidemia is one of the major approaches towards decelerating the atherogenic process. It has been reported that saponins from different sources possess hypolipidemic activity. The objective of this study was to evaluate the antihyperlipidemic activity of saponins of Momordica cymbalaria and Momordica dioica.

**Materials and Methods:**

Chronic hyperlipidemia was induced by feeding cholesterol diet (cholesterol 500 mg/kg suspended in 5 ml of coconut oil) for 35 days to rabbits. The degree of protection was determined by measuring levels of serum TG, TC, HDL-C, LDL-C, VLDL-C and HMG-CoA/Mevelonate. Lovastatin (6 mg/ kg p.o) was used as reference standard. The histopathological changes in the aorta were also studied.

**Result:**

Hyperlipidemia was evidenced by elevated levels of serum TG, TC, LDL-C, VLDL-C. Hyperlipidemic rabbit model exhibited Hypertriglyceridemia, Hypercholesterolemia, and Hyperlipoproteinemia. Treatment with saponins of M cymbalaria (175 mg/kg) and M dioica (55 m/kg) showed significant antihypertriglyceridemic, antihypercholesterolemic, and antihyperlipoproteinemic effect. Saponins of M cymbalaria also reversed the histopathological changes of aorta. Saponins of M dioica did not show significant effect on atherosclerotic lesions in aorta. Saponins of M cymbalaria also decreased the activity of HMG-CoA reductase activity.

**Conclusion:**

Saponins of M cymbalaria and M dioica exhibited antihypertriglyceridemic, antihypercholesterolemic, and antihyperlipoproteinemic activities. Hence Saponins of Momordica cymbalaria exhibited antiatherosclerotic activity.

**662**

**Acanthosis nigricans suggesting insulin resistance and secretion in T2DM patients**

Sharma L^1^, Mathur S K^1^, G Khilnani^2^, Mathur M^1^, Mathur P^1^

**^1^S M S MedicalCollege Jaipur, ^2^RNT Medical College ,Udaipur, India.**

**Objective:**

It is clinically important to predict insulin resistance and secretion in T2DM patients. Acanthosis nigricans and skin tags are cutaneous signs, which suggest underlying insulin resistance and obesity. The relative importance of these in predicting insulin resistance and secretion is not clear. Therefore, we evaluated association of these cutaneous signs with insulin resistance and secretion in T2DM patients.

**Methods:**

One hundred and eighty nine T2DM patients (age 39to 71 years, male to female ratio (103:86) participated in the study. Acanthosis nigricans and skin tags were diagnosed based on clinical criteria. Other physical parameters studied were Body Mass index (BMI) and waist circumference. Laboratory investigations included Fasting and post glucose blood sugar, homeostasis model assessment of insulin resistance (HOMA-R) and secretion.

**Results:**

Presence of acanthosis nigricans was associated with high BMI (*P* = 1.08E-06), HOMA-R (*P* = <.05) and HOMA-β (*P* = < 0.05). Presence of skin tags was associated with higher BMI, triglyceride level. Though it was also associate with higher waist circumference (*P* =>0.05), but the difference was not statistically significant. Similarly, skin tags were also associated with marginally higher HOMA-R and HOMA-β but the difference was not statistically different.

**Conclusions:**

Acanthosis nigricans is associated with not only insulin resistance but also insulin hyper secretion. The high prevalence indicates their use a possible genetic marker in T2DM.

**663**

**Effect of methanolic extract of *Gymnema sylvestre* on animal models of nociception**

Savai J, Kunte S, Sateesh B, Joshi H

**School of Pharmacy & Technology Management, NMIMS University, Mumbai, India.**

**Introduction:**

Leaves of Gymnema Sylvestre (Asclepiadaceae) shown antidiabetic, hypolipidemic, liver tonic, antisweetener, antiinflammatory activities. The present study was done to assess its antinociceptive activity in mice.

**Method:**

Methanolic extract of Gymnema sylvestre (MEG) subjected to TLC and HPTLC. Isopropyl alcohol: chloroform: methanol: acetic acid (5:2:2:0.5) was used as mobile phase. The color was developed with modified vanillin-sulfuric acid reagent and Rf values were determined. Albino mice (25-30 g) were used for acute toxicity study (OECD, 423). MEG was evaluated orally (300 and 600 mg/kg) in formalin induced hyperalgesia and acetic acid induced abdominal constrictions in mice. Phenylbutazone (80mg/kg) was used as reference standard.

**Results:**

TLC analysis showed the presence of gymnemic acids and gymnemasaponins. In oral acute toxicity study, the administration of MEG did not elicit any mortality upto 2g/kg in mice. MEG significantly inhibited acetic acid induced abdominal constrictions (56.25% and 68.75%) at 300 and 600 mg/kg respectively and compared with phenylbutazone (90.62%). In formalin induced hyperalgesia, MEG exhibited more pronounced antinociceptive effect in the chronic phase (20% and 80%) than the acute phase (14% and 29%) at 300 and 600 mg/kg respectively as compared to phenylbutazone(7% and 60%) in acute and chronic phases respectively.

**Conclusion:**

The acute oral toxicity study revealed that the extract is safe and exerts antinociceptive activity in different experimental models of pain in mice at the dose tested. The diverse mechanism of action may involve both central and peripheral pathways.

**664**

**Anti-lithiatic effect of root of *Polycarpea corymbosa* on ethylene glycol induced lithiasis in male albino wistar rats**

Raj Kumar T, Lalitha K G

**Ultra college of Pharmacy, Madurai, India.**

**Introduction:**

Lithiasis is a frequent clinical problem occurring in about 1 to 5% of the population with the recurrence rate of 50 to 80%. The stone diseases are multi-factoral in origin. The available operative and lithotripic procedure are complicated and costly. In this research work, the traditional plant Polycarpea corymbosa of family Caryophyllaceae was chosen and its anti lithiatic effect against ethylene glycol induced lithiasis was observed.

**Method:**

The 80% ethanolic extract of the root of P. corymbosa was screened at 125 and 250 mg/kg p.o doses for antilithiatic activity in male albino wistar rats and the results were summarized based on the (bio-chemical) ionic changes in urine. Lithiasis (stone formation) induced by the oral administration of 0.75 % ethylene glycolated water to adult male albino wistar rats for 28 days. Cystone 750 mg/kg p.o was used as standard drug. The urine Sample (24h) were collected on 7, 14, 21 & 28th days.

**Results and Discussions:**

The ionic chemistry of urine was altered by ethylene glycol, which elevates the urinary concentrations of ions, e.g.: Calcium, Oxalate, Phosphorous there by contributing renal stone formation. Supplementation with ethanolic extract of root of P.corymbosa at 250mg/kg p.o doses significantly (*P*<0.01) reduce the elevated urinary oxalate and calcium ion concentration in urine as compared that of the standard drug, confirming the stone inhibitory effect. Also it elevated the urinary concentration of Magnesium, which is considered as one of the inhibitors of crystallization. The histo-pathological findings also showed signs of improvement after treatment with the extract. All the above observations provide the conclusion that the ethanolic extract of root of *P. corymbosa* is having antilithiatic activity.

**665**

**Antinociceptive property of *Emblica officinalis* gaertn (amla) in diabetic hyperlipidemic rat model of neuropathy**

Premkumar N^1^, Annamali AR^2^, Thakkur RS^1^

**^1^Krupanidhi College of Pharmacy, Bangalore, ^2^Raja Muthiah Medical College, Chidambaram, India.**

**Introduction:**

Increased lipid peroxidation and accelerated ALE (advanced lipoxidation endproducts) formation, possibly catalyzed by hyperglycemia and oxidative stress, may play a critical role in the development of neurovascular complications in diabetes. Herbal treatment is preferred due to lesser side effects and low cost and hence the present investigation is focused on Emblica officinalis Gaertn pretreatment in the setting of diabetichypercholesterolemia associated with diabetic neuropathic pain in rats.

**Methods:**

An experimental model of diabetes mellitus (Streptozotocin 45 mg/kg administered to fructose fed rats) in adult male Sprague-Dawley rats. Ten weeks after the streptozotocin injection the rats were divided into group I (diabetic control), group II (metformin therapy only 500mg/kg), group III (metformin plus simvastatin 20 mg/kg/day), group IV (simvastatin therapy only 20 mg/kg/day), group V (Quercetin only 10 mg/kg/ day) and group VI (Emblica officinalis Gaertn, fresh juice 0.4 g/100 g BW). Blood glucose level, lipid profile, antioxidant status & hyperalgesia (allodynia and non-allodynia) were recorded initially, after 10 weeks of STZ and thereafter every 2, 4 and 6 weeks. The therapy was after 10 weeks of STZ.

**Results and Conclusion:**

Diabetic control exhibited altered blood glucose level, lipid profile, antioxidant status & contributed to hyperalgesia. The therapy attenuated hyperalgesia in the order Emblica officinalis Gaertn, fresh juice > Quercetin 10 mg/kg/day plus simvastatin 20 mg/kg/day > Quercetin only 10 mg/kg/day > metformin plus simvastatin 20 mg/kg/day > simvastatin therapy only 20 mg/kg/day > metformin therapy only. The significant activity of Emblica officinalis Gaertn is correlated with its hypoglycemic, hypolipidemic and antioxidant activity.

**666**

**Effectiveness of new gaba analogues against febrile seizures in an immature rat model**

Katiyar S, Viyogi S, Reddy A S K, Ragavendran J V, Sriram D, Yogeshwari P

**Birla Institute of Technology and Science, Pilani, Rajasthan, India.**

Febrile seizures are the most common type of developmental seizures, affecting up to 5% of children. The purpose of our study was to evaluate newer GABA analogues designed on the basis of pharmacophore hybridization, in combating febrile seizures. Three sets of test compounds (GSC, SKI and SVP series) were tested for activity. 21 days old rat pups were used for study. Rats were administered intra-peritoneally single dose of vehicle (30%v/v PEG 400), test compounds (300 mg/kg) and standard drug (Lamotrignine 100 mg/kg) 30 min prior to hyperthermic exposure. Exposure to hyperthermia was done by placing the rat pups in controlled temperature (45°C) water bath. The control or normal rat pups were maintained at a temperature of 37°C. The pups were placed in the water unrestrained for 5 min or until a seizure occurred (whichever was shorter). The pups were immediately removed from the water at the first sign of seizure onset. Both the time of onset and the duration of seizure were recorded and compared with the hyperthermic control group. Of the total of twenty-six compounds screened, eleven compounds prolonged the time of onset of seizures and also shortened the duration of seizures. In conclusion, lipophilic analogues of GABA apart from increasing the BBB penetrating ability of GABA, could also serve to increase the endogenous concentration of the neurotransmitter by multifarious mechanisms and can be a potent therapeutic approach for the treatment of febrile seizures.

**667**

**Effect of sodium cromoglycate on ischaemic reperfusion injury in isolated rat heart**

Goswami HG, Savaliya JT, Shah GB

**K. B. Institute of Pharmaceutical Education and Research, Gandhinagar, India.**

**Introduction:**

In the present study, sodium cromoglycate, a mast cell stabilizer was studied in ischemic reperfusion injury in isolated rat heart.

**Methods:**

Isolated perfused rat hearts were perfused with 100 mg/ml of sodium cromoglycate in Kreb’s Henseleit (K-H) solution for 20 min. Later the preconditioning procedures were initiated which consist of four brief ischaemia (5min.) – reperfusion (5min.) cycles. The ischaemic period was than extended for 9min. followed by reperfusion period of 15 min. to produce ischaemic reperfusion injury. The perfusate after during preconditioning and after ischaemic reperfusion injury were collected to estimate mast cell peroxidase (MPO), creatine kinase (CK), lactate dehydrogenase leakage (LDH) and glutamate oxaloacetate transminase (GOT) levels. After the completion of this procedure the heart was homogenized and tissue levels of catalase activity, malondialdehyde, superoxide dismutase and glutathione levels were measured. In another group of rats, the preconditioning procedures were omitted and other procedures were carried out as mentioned earlier. In the third (control) group of rats except for sodium cromoglycate administration all procedures were carried out.

**Results:**

In sodium cromoglycate treated rat hearts, significant lower activites of lactate dehydrogenase, creatine kinase, mast cell peroxidase and glutamate oxaloacetate transminase were observed as compared to control group. Also tissue level of catalase, malondialdehyde content, glutathione and superoxide dismutase were found significantly higher compared to control.

**Conclusions:**

Hence, the results of our study provide some new information that cromoglycate pretreatment provides significant protection against ischaemic-reperfusion injury by virtue of the mast cell stabilizing property.

**668**

**Newer approach for treatment of dry eyes**

Bhavsar A^1^, Bhavsar S^2^, Jain S^3^,^1^

**Sat Kaival college of Pharmacy, Sarsa, ^2^S.K. Hospital, Karamsad, ^3^L.M. college of Pharmacy, Ahmedabad, India.**

Dry eye is an extrermely common and often unrecognized disease. Due to multifactorial and elusive etiology, it is often challenging to treat dry eye. Recent studies suggest that apoptosis may play a key role in pathogenesis of dry eye. Conventional treatment by lubricating eye drops gives only symptomatic relief but does not treat the underlying cause of the disease. To overcome this problem, few newer drugs have emerged which might be effective because of their anti inflammatory property, one of them is topical chloroquine phosphate. The aim of the present study is to compare the safety & effficacy of topical chloroquine phosphate with conventional treatment of dry eye. Patients of dry eye with tear film break up time less than 10 seconds are considered for randomised case control study. Topical chloroquine phosphate 0.05% preservative free solution was used twice a day for 21 days. A standardised questionarie of Ocular Surface Disease Index (OSDI) was given to each patient before the study and every week thereafter. Other parameters like Tear Film Break Up Time (TBUT), Schirmers test, Fluorescine staining and Rose Bengal staining of ocular surface were examined at regular intervals before & during the study. The initial results of our study showed significant improvement in OSDI score & TBUT in patients treated with topical choroquine eye drops. Chloroquine is known to inhibit metalloproteases liberated by macrophages, neutrophils and dead or dying cells. The anti inflammatory property of chloroquine is because of its antimetalloprotease action. It can therefore protect the immature epithelial surface from various insults, which cause apoptosis & inflammatory reaction which in turn causing and aggravating dry eye problem. Hence, it can be suggested that topical choroquine phosphate can be a better option for the treatment of dry eye.

**669**

**Anti-diabetic herbal formulation (mersina) attenuate progression of renal damage in streptozotocinnicotinamide induced diabetic rats**

Sateesh B, A Veeranjaneyulu

**School of Pharmacy & Technology Management, NMIMS University, Mumbai, India.**

**Introduction:**

Diabetic nephropathy is among common causes of renal failure, is associated with oxidative stress and chronic hyperglycemia. Role of herbs in diabetic complications including nephropathy are reported. Present study is done to investigate the role of “Mersina” (antidiabetic Ayurvedic formulation with antihyperglycemic and antioxidant contents) in diabetic animals.

**Methods:**

Albino rats, wistar strain (220-250g) are used. Diabetes was induced by streptozotocin (65 mg/kg) and nicotinamide (110 mg/kg) intraperitoneally. Mersina (300 and 600mg/kg), administered orally daily for a period of 40 days to diabetic animals. Plasma creatinine and blood urea nitrogen (renal function tests), plasma glucose, triglyceride and cholesterol (metabolic function tests), body weight, urine volume, urinary creatine, ketone, glucose and protein levels were monitored on every 10th day over a 40-day period of the experiment. Glycosylated haemoglobin levels were monitored on 0 day and on 40th day of the experiment. On 40th day, animals were sacrificed and the kidney weight was determined. Renal hypertrophy was assessed.

**Results:**

Significant Improvement in renal and metabolic parameters were observed in Mersina treated diabetic rats compared to control rats (*P*<0.005). Body weight, urine volume, urinary creatine, ketone, glucose and protein levels were significantly improved in Mersina treated diabetic rats compared to control diabetic rats (*P*<0.005). Renal hypertrophy was significantly higher in diabetic controls as compared to non-diabetic controls. ‘Mersina’ partially but significantly (*P*<0.05) prevented renal hypertrophy as compared to diabetic controls.

**Conclusion:**

Results indicate that the ingredients of Mersina, synergestically attenuated the progression of kidney damage in streptozotocin-nicotinamide induced diabetic rats.

**670**

**A study of the anti-inflammatory activity of the ethanolic extract of *Spilanthes acmella* on experimental animal models**

Dutta S, Das S

**Assam Medical College, Dibrugarh, India.**

**Introduction:**

The inflammatory process is the response to an injurious stimulus. It occurs in three distinct phases-acute, subacute and chronic. Spilanthes acmella, an annual herb found in tropics and belonging to family Compositae possess medicinal properties. It is used in toothache, rheumatism, periosteitis etc.

**Methods:**

Fresh aerial parts of the plant was collected, air-dried, powdered and percolated for 48 hrs in ethanol. Acute toxicity test was done according to OECD guidelines. 4 groups of animals of either sex, weighing 150-200 g of the species Rattus norvegicus are taken for the study (n=6). Group I taken as normal control (3% gum acacia in 10 ml/kg body weight), Group II as test group (SAE 250mg/kg body weight), Group III as test group (SAE 500mg/kg body weight) and Group IV as standard (Aspirin 100mg/kg body weight). The animals were studied for acute inflammation by carrageenan induced rat paw edema, subacute inflammation by granuloma pouch method and chronic inflammation by Freund’s adjuvant induced arthritis method.

**Results:**

In acute inflammation there was significant inhibition of paw edema in group II, III and IV in comparison to Group I (*P*<0.05). In subacute inflammation, there was significant inhibition of exudate formation in Groups II, III and IV in comparison to Group I (*P*<0.05). In chronic inflammation there was significant inhibition of increase in paw edema and inhibition of weight reduction in Groups II and III (*P*<0.05). Downregulation of arthritic index was also significant in Groups II,III and IV in comparison to Group I (*P*<0.05).

**Conclusion:**

The ethanolic extract of Spilanthes acmella has significant anti-inflammatory activity.

**671**

**Effect of sertraline against 3-nitropropionic cid induced behavioral, oxidative stress and mitochondrial dysfunctions in rat brain**

Kumar P, Kumar A

**Panjab University, Chandigarh, India.**

**Background:**

HD is an inherited progressive neurodegenerative disorder characterized by progressive movement, psychiatric and cognitive disturbances. 3-NP (mitochondrial toxin) produces age-dependent oxidative linked striatal damage, responsible for HD particularly in middle age population. The objective of the present study was designed with an aim to explore the possible neuroprotective mechanism of sertraline against 3-nitropropionic acid (3-NP)-induced behavioral, biochemical, mitochondrial alterations in discrete areas of rat brain.

**Materials and Methods:**

3-NP (10 mg/kg, ip) was administered for 14 days. Various behavioral parameters (locomotor activity, body weight, rotarod activity performance), followed by oxidative damage (elevated levels of lipid peroxidation, nitrite concentration, depletion of antioxidant enzyme levels) and mitochondrial dysfunction (Complex –I, II, II and IV) was assessed in striatum, cortex and hippocampus.

**Results:**

3-NP (10 mg/kg, ip) administration for 14 days significantly induced HD like symptoms in rats as indicated by reduced locomotor activity, body weight, rotarod activity performance and significant oxidative damage (elevated levels of lipid peroxidation, nitrite concentration, depletion of antioxidant enzyme levels) and mitochondrial dysfunction (Complex –I, II, II and IV) in striatum, cortex and hippocampus. Treatment with sertraline (10 mg/kg) significantly improved behavioral activity as compared to control (*P*<0.05). However, sertraline (5 and 10 mg/ kg) significantly attenuated oxidative damage and mitochondrial enzyme dysfunctions as compared to 3-NP treated group (*P*<0.05).

**Conclusion:**

The present study suggests the possible neuroprotective and antioxidant effect of sertraline against 3NP induced alterations in animals.

**672**

**A randomized controlled clinical trial of duloxetine as compared to imipramine in somatization disorder**

Dubey AK, Pandey SP, Raichandani OP

**NSCB Medical College, Jabalpur, India.**

**Objective:**

To study the efficacy and tolerability of Duloxetine, a dual reuptake inhibitor, in Somatization disorder and to compare it with Imipramine, a tricyclic antidepressant.

**Materials and Methods:**

A randomized, prospective, double-blinded, parallelgroup comparative study between Imipramine and Duloxetine was conducted on 60 patients of Somatization disorder, over a period of one year, half of whom were prescribed 150mg Imipramine and half 40mg Duloxetine daily, in two divided doses over a period of 45 days, with a lead-in period of 7 days. They were assessed at baseline and then fortnightly using the CGI Scale.

**Results:**

The total improvement in CGI score of patients on Imipramine and Duloxetine is comparable (38.73% vs 46.55%). Duloxetine shows a slightly better effect than Imipramine but the difference in their efficacy is not statistically significant (*P*>0.05). While considering the tolerability of the two drugs, it was found that except for nausea, which was reported more by the patients on Duloxetine, the number of patients reporting the side effects and the variety of side effects were more in the patients who were on Imipramine, which accounted for a statistically significant difference in drop-out rates.

**Conclusion:**

Both Imipramine and Duloxetine show comparable efficacy in the treatment of Somatization disorder. However the side effect profile of Duloxetine is better than Imipramine leading to significantly lower attrition rates.

**673**

**Topiramate prevents olanzapine induced metabolic alterations in schizophrenia**

Narula PK, Rehan HS, Unni KES

**Lady Hardinge Medical College and associated Smt. S. K. Hospital, New Delhi, India.**

**Introduction:**

Olanzapine, a second-generation antipsychotic is used for the treatment of schizophrenia. Its use is associated with weight gain, hyperglycemia and dyslipidemia leading to increased cardiovascular morbidity. Recently, Topiramate has been used as an adjunct therapy in patients with obesity, hypertension and diabetes to decrease weight. This activity of Topiramate could be used to prevent Olanzapine induced weight gain and metabolic abnormalities in schizophrenia.

**Methods:**

We assessed the efficacy of Topiramate as an adjunct treatment in schizophrenia patients (ICD 10 Diagnostic Criteria) by administering either Olanzapine (5-20 mg/day) (n=34) or Olanzapine + Topiramate (100 mg/day) (n=33) for 12 weeks. Weight, blood glucose, lipid profile and PANSS (Positive and Negative Syndrome Scale) were recorded at baseline and at 12 weeks.

**Results:**

After 12 weeks, in the Olanzapine group, there were significant increase (*P*<0.001) in weight, glucose, cholesterol and triglyceride levels. In the Topiramate + Olanzapine group, there was a mean weight loss of 1.27 ± 2.28 kg (*P*<0.01) and a decrease in glucose, cholesterol and triglyceride levels (1.52 ± 5.3, 1.94 ± 10.48, 2.24 ± 8.12 mg% respectively). Topiramate prevented an increase in weight and adverse metabolic changes when combined with Olanzapine. Significant clinical improvement (reduction in PANSS scores) (*P*<0.001) was observed in both the treatment groups. Four patients in the Topiramate group had difficulty in concentration and psychomotor slowing.

**Conclusion:**

Our study demonstrates a benefit of Topiramate + Olanzapine combination on the patients’ symptoms and to prevent Olanzapine induced weight gain, hyperglycemia and dyslipidemia in schizophrenia.

**674**

**Antihyperglycaemic activity of *Cinnamomum macrocarpum***

S Srinivasan^1^, P Sureshkumar^1^, A Benno susai vijayakumar^2^, V Sudarshana Deepa^1^

**^1^Anna University, Tiruchirappalli, ^2^St. Joseph’s college, Tiruchirappalli, India.**

Assessment of antihyperglycaemic activity of Cinnamomum macrocarpum (CM) on the streptozotocin (STZ) induced diabetic rats was carried out in the present study. The effect of 70% ethanolic bark extract of CM on fasting blood sugar levels (FBS) and serum biochemical parameters was investigated. When rats were pretreated with the bark extracts of CM, significant antihyperglycaemic activity was noted at a dose of 100mg/kg bodyweight of the rat. There was a considerable increase in blood sugar level in the STZ induced rats almost five fold when compared with normal control rats. There was a significant antihyperglycemic activity in the CM pretreated rats as it reduces the blood glucose level from (309 ± 5.16 mg/dl) to 69 ± 9.24 mg/dl. The substantially elevated serum hepatic enzyme markers due STZ induction in the experimental rats such as SGOT, SGPT, ALP, LDH, GGT and Lipid hydroperoxides levels were restored towards normalization only in CM pretreated groups. Further Histopathological studies of the liver and pancreas of showed that the extent of damage was comparatively lesser in the CM treated groups when compared to the diabetic rats. The necrotic damage is suppressed by the plant extracts. The pre treatment with these plant extract shows a considerable recovery from the damage.

**675**

**Anti-inflammatory activity of leaf extract of *Ricinus communis***

Chakraborthy GS

**School of Pharmacy and Technology Management, SVKM’S, NMIMS University, Shirpur Campus, Dist Dhulia, Maharashtra- 425 405, India.**

**Objective:**

To evaluate the acute and chronic anti-inflammatory properties of leaf extracts of Ricinus communis in rats.

**Materials and Methods:**

The methanolic extracts of Ricinus communis were extracted by using petroleum ether, ethyl acetate and n-butanol. The anti-inflammatory profiles of these extracts were investigated on the basis of paw edema induced by carrageenan. The n-butanol (most potent) was further assessed through acute inflammatory models by formalin and histamine. The chronic anti-inflammatory and the ulcerogenic activities of chloroform fraction were also examined.

**Results:**

On oral administration of n-butanol fraction (100 mg/kg) caused a maximum inhibition of about 46% in paw edema induced by carrageenan. The n-butanol fraction also exhibited acute inflammatory activity on paw edema induced by histamine (49.51%), formalin (38.56%). In chronic inflammation model, this extract showed maximum inhibition of 63.26% on eighth day of treatment. The ulcerogenic assessment showed the ulcer indices after oral administration with chloroform fraction were almost nil and 0.2± 0.1 for 100 mg/kg dose.

**Conclusion:**

On the basis of above findings, it may be concluded that Echinacea is a potent anti-inflammatory as well as antiarthritic agent which usually blocks the pathways of histamine. The results obtained were statistically significant, thus the exact active constituent responsible for the activity may be traced for the betterment of mankind.

**676**

**Evaluation of isoflavone effect on thyroid economy and autoimmunity in oophorectomised women: A randomized double-blind placebo controlled clinical trial**

Mittal N^1^, Hota D^1^, Dutta P^1^, Suri V^1^, Marwah RK^2^, Chakrabarti A^1^

**^1^PGIMER, Chandigarh, ^2^INMAS, New Delhi, India.**

Phytoestrogens by virtue of their potential to act as selective estrogen response modifiers (SERMs) have received attention as alternatives to hormone replacement therapy for post menopausal women. However, soy isoflavones, the most common form of phytoestrogens, have been implicated to interfere with thyroid function. Moreover, there is no data regarding their effect on thyroid function and autoimmunity in oophorectomised women. This randomized, double blind, placebo – controlled trial evaluated the effect of soy isoflavones (75 mg/day for 12 weeks) on thyroid function (serum free T3, free T4, TSH, TBG) and auto-immunity (anti – TPO antibody titres) in 34 women who had undergone bilateral oophorectomy. The outcomes were assessed at baseline and at 12 week after randomization to either group. The two study groups were comparable in terms of demographic, clinical characteristics and thyroid profile at baseline. There was a significant decrease in free T3 levels in the group receiving isoflavones (4.01 ± 0.08 and 3.76 ± 0.13 pmol/L at baseline and 12 weeks respectively; *P* = 0.009) however isoflavones did not significantly affect other parameters during study period. Also intergroup comparisons for various parameters were not different significantly at 12 week. There were no serious adverse events reported and the proportion of patients experiencing adverse events was similar between two groups (*P*=1.00). Isoflavones did not adversely affect thyroid function in oophorectomised women compared to placebo. Modest effect on free T3 without any other effect on thyroid function might be considered clinically unimportant.

**677**

**Snake bite severity assessment: A tool for the management of snake envenomation**

Khan Faisal, Peerally S, Saghir Z, Menon R, Kishore S

**Manipal College of Pharmaceutical Sciences, Manipal University, Manipal, India.**

Snakebite is a major medical problem in tropical countries and is responsible for 50,000-100,000 deaths each year. Assessment of clinical severity can help to decide the most appropriate therapy and improve effectiveness of therapy. Hence a study was carried out to assess the severity and treatment of snakebite in a tertiary care hospital of South India. A prospective cross sectional study was carried out between 2003 to 2006 to assess the clinical characteristics, severity, treatment and outcome of snakebite patients. Various determinants affecting severity, the treatment and outcome were analysed. A total of 252 snakebite patients were admitted with more males n=154 (61%) than females n=98 (39%). Majority of snakebite envenomation were unknown 105 (41.7%) followed by cobra 64 (25.4%), viper 60 (24%), Krait 15(5.9%), non poisonous snakes (n=5) and King cobra (n=3). Majority of the patients developed ptosis 43 (31.2%), breathing difficulty 37 (26.8%) and cellulitis 94 (68.1%). Majority of patients N=169 (67.1%) had a severity grade between 2 and 3; 95 (37.7%) of them had grade 3 severity denoting severe life threatening symptoms. A significant (*P*<0.001) association was observed with severity grades, type of snakes and outcome. A total of 2994 ASV vials were employed, 66 (26.3%), patients developed anaphylactic reactions and hence were administered corticosteroids and antihistamines injections. Outcome analysis showed that 27 (10.7%) patients died, 26 patients worsened, while 199 cases improved at discharge. Severity grading of snakebite patients correlated with outcome and hence recommended as an important tool in determining the extent of treatment required.

**678**

**Pharmacogenomics: Uses and benefits in clinical practice and pharmaceuticals**

Shukla A

**V.M.C. Pondichery, India.**

**Objective:**

Pharmacogenomics is pharmacology with genomics to see the effect of drug and genetic makeup. On personalize drug efficacy and safety needed for individual response to medication.

**Methods:**

This is very wide field, it includes 1) Basic Research 2) Clinical - Efficacy Improvement in patient, GX Testing, Effectiveness PGX Clinical Relevance, Effective and cost consequences 3) Clinical Practice Clinical Use of PGX, Implementation and acceptance of PGX, PGX testing like 6 – mercaptopurine, Irinotecon-Warfarin, Trastuzumb, Nortryptilin. The Institution Must Have Following Services. 1. DNA & Tissue banking, 2. Genotyping & Genomic Services, 3. Good lab for DNA isolation, 4. Pharmacogenomics: Clinical services with (a) Pharmacokinetics and Pharmacodynamics analysis, (b) Pharmacokinetics proteomic analysis, (c) GLP data analysis, (d) Clinical Coordination for Toxicity drug efficacy.

**Result:**

The individual drug therapy includes test operating at protein, metabolized or biomarker affected by genetic variation like single nucleotide polymorphism, insertion, deletion, micro satellite variance in copy number and both heritable and non-heritable mutations are considered and immunohistochemical test such as that HER/neu are also considered for PGX test, and also single dose test for poor metabolizer and ultra metabolizer.

**Conclusion:**

The variation in drug response is safer and more effective by use of PGX testing and permitting individualized therapy. At the same time, while genetic testing can provide answers for developing better drugs, making better diagnosis, and delivering better care, it raises question’s too.

**679**

**Non alcoholic fatty liver disease and role of indian medicinal plants: Developing a suitable model for evaluation of efficacy of natural molecules**

Kulkarni K^3^, Patil S^1^, Fursule R^2^

**^1^R. C. Patel Institute of Pharmaceutical education and Research, ^2^H. R. Patel Women’s School of Pharmacy; ^3^SVKM’S NMiMS University SPTM, Shirpur Campus, India.**

Non alcoholic fatty liver disease or steatohepatitis is most prevalent in western population. It progresses from steatosis to steatohepatitis and cirrhosis of liver. The biochemical changes seen here may reflect as alcoholic liver disease. Dr. J. Ludwig from the Mayo Clinic made observation that certain individuals who share common features of obesity, diabetes and elevated cholesterol had a liver disease that closely resembled alcoholic liver disease. However, these patients did not have evidence of alcohol consumption, coined the term nonalcoholic steatohepatitis (NASH). The prevalence of each is presumably much higher in obese and diabetics. It is associated with primary insulin resistance. Insulin resistance can also surface early in life when it is due to congenital genetic abnormalities in the insulin receptors; it becomes evident later in life as a result of acquired obesity. Insulin resistance leads to changes in the metabolism of glucose and lipid in the liver, muscles and adipocytes. A sedentary lifestyle and a diet rich in carbohydrates and fats also promote insulin resistance. Elevated LDL cholesterol and triglycerides are also associated with insulin resistance. Resultant increased infiltration and accumulation of triglycerides into the hepatic canaliculi cells. In addition, Leptin resistance may contribute to the development of NASH. Number of medicinal plants has shown hepatoprotective activity against carbon tetrachloride, acetaminophen and aflatoxin induced hepatotoxicity. It is necessary to develop the model simulating NASH in terms of liver biochemistry and biomarkers with specialized diet containing high concentration of fats and carbohydrates in experimental animals. This would be used to evaluate the efficacy of hepatoprotective agents in NASH.

**680**

**Management of cancer pain in advanced cancer patients**

BIsht M, Dhasmana DC, Saini S

**Himalayan Institute of Medical Sciences, Dehradun, India.**

Pain is the most persistent and incapacitating symptom of advanced cancer. Despite existing WHO guidelines for pain management, many patients with cancer pain receive inadequate relief. An observational study was carried out to investigate the pharmacological management of pain in advanced cancer patients receiving palliative care in a tertiary care hospital of Uttarakhand. 100 patients with advanced cancer irrespective to the tumor site were included and followed up for a period of two months in the study and their pain severity (calculated by Visual analog scale and present pain intensity scale), degree of relief provided with analgesic drugs along with other prescription details were recorded. The analgesic treatments received by the patients were analyzed in terms of their dosage, duration and route. The adequacy of pain management using the pain management index was assessed, which revealed whether the prescribed analgesics were congruent with pain severity. The results revealed that 95% of patient reported pain and 91 out of 95 (95.79%) rated the pain as substantial (a score of 5 or higher). Analgesic drug therapy was prescribed to the patient according to the WHO analgesic ladder. Of the patients with pain 28.42% received WHO level 1 drug, 65.26% WHO level 2 drug and only 6.32% received morphine. 4.25% of patients did not receive any analgesic agent. 66% of patient were not given adequate analgesic treatment as assessed by the pain management index. This study suggests that the cancer pain management was insufficient at the investigated institute in Uttarakhand.

**681**

**Some observations on poisoning cases reported at a Tertiary Hospital at Jaipur**

Mathur M, Gupta B M, Saini M K, Sharma L

**S.M.S.Medical College, Jaipur, India.**

**Introduction:**

Acute cases of poisoning are commonly reported at the Emergency of the S.M.S. Hospital. A proposal to establish a Poison Information and Toxicological Research Centre in the State has been mooted. In order to prepare a data base a preliminary retrospective survey was conducted. Some observations are reported here.

**Methods:**

Data was collected from cases records available with the Forensic Department of the Hospital. The data was analyzed for various aspects of poisoning with a view to determine specific patterns.

**Results:**

The two major category of poisoning noted were OPC 13.2% (14/106) and Celphos 17.9 % (19/106). 28% were dead (rural 63%; urban 37%) by the time they reached the hospital and in about 32% the nature of poison could not be determined. OPC poisoning was common in males (78.6%) and in the rural population (64.3%). In celphos poisoning, no significant sex or rural/ urban difference was noted; however it was consumed by a high percentage of females (6/8) from rural areas. Other characteristics noted were that poisoning was accidental in only 13%; with suicidal intent 50% including nine students. Celphos was taken with suicidal intent by 17 of the 53 persons while 10 took OPC. Another interesting feature of the data analysis was that poisoning was most common in population below 35 years of age (91 out of 106 cases).

**Discussion:**

While the data is from a single centre and small number, it depicts a discernible pattern which stresses the importance of a PIC in providing important clues to the treating physician and to the public on first aid measures which can help save valuable lives.

**682**

**Effect of α-tocopherol supplementation on arsenic and/or fluoride induced changes in brain biogenic amines level and glutathione linked enzymes: Biochemical and spectroscopic approach**

Mittal M, Flora SJS

**Defence Research & Development Establishment, Gwalior, India.**

Arsenic and fluoride are common water pollutant. In the present study we aimed at determining the combined toxic effects of arsenic and fluoride on (i) brain biogenic amines, (ii) glutathione linked enzymes, (iii) structural integrity of DNA and, their response to α-tocopherol. Male mice were exposed to arsenic (50 ppm) and fluoride (50 ppm) individually and in combination for 10 weeks and co-administered with vitamin E (5 mg/kg, i.m., alternate days). Arsenic caused a significant decrease in blood δ-aminolevulinic acid dehydratase (ALAD) activity followed by increased reactive oxygen species (ROS) level. Individual exposure to arsenic and fluoride significantly decreased the concentration of nor epinephrine (NE), dopamine (DA) and 5-hydroxytryptamine (5-HT) however; did acetyl cholinesterase and monoamine oxidase activities increase during fluoride exposure alone. These changes were accompanied by increased ROS and TBARS level and decreased SOD activity. Glutathione metabolism was also altered by arsenic and fluoride exposure, indicated by decreased GSH: GSSG ratio, decreased glucose 6-phosphate dehydrogenase activity and increased activity of glutathione S-transferase (GST) and glutathione peroxidase (GPx). Combined exposure to these toxicants had no additional effect except for a more pronounced increase in AChE, MAO, SOD and catalase activities. Arsenic and fluoride affected DNA structure and leads to some structural deformities. Spectra revealed less pronounced toxicity during combined exposure as the characteristic peaks of cytosine and α-helical structure of DNA were observed in normal and arsenic plus fluoride exposed animals. Supplementation of vitamin E was able to ameliorate free radicals from the body thereby minimizing the condition of oxidative stress. It can be concluded from the study that combined exposure to arsenic and fluoride had few synergistic effects which responded favorably to concomitant administration of vitamin E.

**683**

**Palonosetron induced anaphylaxis: Need for caution**

Saravana Perumal S, A. Goyal, B.M. Padhy, Y.K.Gupta, M. Singhal, V. Raina

**All India Institute of Medical Sciences, New Delhi, India.**

**Introduction:**

Serotonin subtype 3 (5-HT3) antagonists are effctive for prevention of chemotherapy induced emesis. Ondansetron, granisetron and palonosetron are commonly used 5HT3 antagonists in India. Palonosetron a relatively new drug has stronger receptor binding affinity.

**Objectives:**

The study was aimed to evaluate the frequency of, use of palonosetron and, serious adverse event with palonosetron in prevention of chemotherapy induced emesis.

**Methods:**

Patients attending the day-care unit of Dr. B.R. Ambedkar’s Institute Rotatry Cancer Hospital, AIIMS who received 5-HT3 antagonist before chemotherapy were monitored for adverse events.

**Results:**

A total of 1079 subjects received 5HT3 antagonists during the period of one month (Jan2008). Palonosetron was used as antiemetic premedication in 15.8% patients. The other 5-HT3 antagonists used were ondansetron (74.6%) and granisetron (9.6%). One SAE was reported at the zonal pharmacovigilance centre with the use of intravenous palonosetron. A 40 year old female with relapse of carcinoma breast was admitted to receive systemic adjuvant chemotherapy. She was a known case of allergic dermatitis and also had allergy to detergents containing caustic soda. Within a few seconds of intravenous administration of 0.25mg palonosetron, patient developed anaphylactic shock. Normal saline, intravenous adrenaline, dexamethasone, hydrocortisone, chlorpheneramine and supplemental oxygen were used to revive the patient and she was kept under observation for 24hrs. Causality analysis carried out using the WHO-UMC criteria revealed a certain causal relationship of the reaction with palonosteron.

**Conclusion:**

Of all the patients receiving 5-HT3 antagonists, only one serious adverse event was observed with palonosetron indicating the need of caution with its use especially in patients with known history of allergies.

**684**

**Six sigma concept in health care**

Sukhlecha A

**M P Shah Medical College, Jamnagar, Gujarat.**

Six Sigma is a statistical concept that measures a process in terms of defects—at the six sigma level, there are only 3.4 defects per million opportunities The key steps in the six sigma improvement framework are Define - Measure- Analyze - Improve - Control Tips for Improving Patient Safety:

Develop clear policies and protocols for patient safety aligned with national guidelines and organizational objectives.
Provide regular communication on patient safety initiatives throughout the organization.
Establish a culture which understands that quality is part of an enterprise-wide endeavor. Efforts should not be relegated to a single person or department.
Encourage transparency through a non-punitive system for tracking errors and near-misses.
Ensure technology is an appropriate enabler for quality (rather than a hindrance) by addressing the age, condition, utilization and relevant training involved with specific equipment, such as heart monitors.
Make sure patient safety projects are well defined with appropriate parameters and targets.
Support and implement patient safety education for staff, physicians, patients and families.

Two important criteria in selecting a project for Six Sigma are effort required and probability of success. A quick and easy way to remember the major aspects of a successful project is the acronym SMART-Specific, Measurable, Achievable, Realistic, Time bound.

**685**

**Analgesic and anti-inflammatory activity of ficus bengalnensis**

Darade S, Alai M, Patel E, Gadiya P, Gaikwad S, Gulecha V

**SSDJ College of Pharmacy, Neminagar, Chandwad, Nasik.**

The effect of PHF was investigated in experimental models of pain and inflammation. Analgesic activity of MFB (10, 30, and 100mg/ kg, p.o.) was studied in mice using acetic acid induced writhing, tail immersion method and hot plate method. Anti-inflammatory activity of MFB (10, 30, and 100mg/kg, p.o.)was studied in rats using carrageenan induced hind paw edema and formalin induced rat paw edema method. MFB (10, 30, and 100mg/kg, p.o.) significantly (p<0.05) reduced the number of writhing, increased latency to flick tail in tail immersion method and elevated the mean basal reaction time in hot plate method. MFB (10, 30, and 100mg/kg, p.o.) ignificantly (p<0.05) inhibited carrageenan induced hind paw edema and formalin induced rat paw edema.

**686**

**Osteopontin: Isolation, purification and characterization from buffalo milk**

Mathur DS^1^, Sharma S^1^, Sinha M^1^, Gupta YK^1^, Sangwan RB^2^, Kumar R^2^, Mann B^2^

**^1^All India Institute of Medical Sciences, New Delhi, ^2^National Dairy Research Institute, Karnal, India.**

**Introduction:**

Osteopontin (OPN) is a multifunctional bioactive glycosylated phosphoprotein containing specific domains for interaction with calcium, heparin, CD44, and integrins. OPN is involved in biomineralization, inflammation, cell binding, immunity acquisition, fertility, renal stone inhibition and protection against microbial infections. Plasma OPN levels are associated with a wide range of heart, lung, liver and other diseases. Treatment method of ectopic calcification using OPN has been patented. It is used as a marker in allergen immunotherapy, hepatitis and cancer. OPN has been identified in cells, tissues and physiological fluids of various species. Objective: Milk is an easily accessible source of OPN and present study was undertaken to isolate, purify and characterize it from buffalo milk.

**Materials and Methods:**

Pooled buffalo milk sample was taken from the cattle yard of National Dairy Research Institute, Karnal, Haryana (India). Protein was isolated by ionexchange (DEAE-Sephacel) chromatography followed by purification using hydrophobic interaction and hydroxyapatite chromatography respectively. Purified protein was dialysed and freeze-dried. Dot blot analysis for detection was carried out on nitrocellulose membrane using anti-osteopontin-mouse (developed in goat), rabbit-anti-goat- IgG-HRP conjugate and standard osteopontin-mouse (recombinant) for comparision.

**Results:**

N-terminal sequence of the molecule was determined to be RIAVICFCLLGI. ~8 mg of OPN was yielded from 1L of raw buffalo milk. Protein was shown to have a high antioxidant activity. Alcian blue-silver staining gave enhanced band intensity over coomassie blue of the SDS-PAGE gels. Purified protein was also characterized for amino acids, phosphorus and carbohydrates.

**Conclusion:**

Osteopontin is present in buffalo milk. However, further studies are required to explore additional diagnostic and therapeutic potential of OPN.

**687**

**Use of gold nanorods for laser-induced photothermal therapy**

Dinda A. K.^1^, Prashant C. K^1^, Rajiv Kumar^1^, Amarnath Maitra^2^, M.Samim^2^

**^1^All India Institute of Medical Sciences, New Delhi, ^2^Delhi University, New Delhi, India.**

Plasmon-resonant gold nanorods with near infra red (NIR) absorption bands are demonstrated as agents for nanophotothermolysis. Gold nanorods were synthesized by a simple process. Wistar rats were injected with the gold nanorod solution or the gold nanorod solution was painted on a patch of skin. An infra-red laser was directed at the point of injection or on the painted skin surface. Rats injected with saline or painted with saline solution were considered as negative controls. After defined time points of exposure to laser, the rats were sacrificed and the skin and tissue at the point of application of the laser was cut out, fixed in formalin and embedded in paraffin. Sections from the paraffin block were stained with haemotoxylin and eosin and observed under a light microscope. The skin and tissue sections from rats injected or painted with the gold nanorod solution showed evidence of injury due to burning caused by the laser absorption of near infra red light by the gold nanorods and their subsequent heating. The tissue appeared inflamed with infiltration of neutrophils. No such phenomenon was observed in sections obtained from the negative control rats. Hence this method of gold nano-rod induced photothermolysis holds promise for treatment of cancer, even in cases where the cancer is deep within the tissue and surgical resection is not possible. This technology may be safer and cost effective in comparison to conventional radiotherapy with less side effects.

**688**

**Lead induced oxidative stress countered by rhizome powder of *Curcuma longa* (turmeric)**

Kala Kumar B, Hyndavi L, Reddy A G

**College of Veterinary Science, Hyderabad, India.**

Broilers are subjected to high amount of stress due to pollutants, mycotoxins, vaccination,physical handling etc., One of the mechanisms of Lead toxicity is by induction of free radicals. Free radicals cause cell necrosis through lipid, protein peroxidation and nucleic acid alkylation. Four groups (n=20) of broiler chicks were assigned into sham, lead control treated in feed @ 2.5ppm, turmeric control fed @ 5g /Kg of feed, and lead and turmeric fed groups for 4 weeks. Antioxidant defence profile, immune profile were assessed and HPE of the organs was done. Lead control group showed a significant decrease in antioxidant and immunity profile. Histopathology of liver and kidney revealed toxic changes as compared to sham and turmeric control groups. Simultaneous feeding of turmeric has alleviated the detrimental effects of lead. There was no significant change in the antioxidant and immune profiles. The histopathology of the liver and kidney did not reveal any toxic changes. These findings indicate that feed supplementation of turmeric would serve in prophylaxis of oxidative stress.

**689**

***In vivo* efficacy of RBx 33B4088C in animal models**

Raj Kumar S, Sinha S, Siddiqui MA, Gupta S, Kumar N, Sarma P K, Salman M, Ray A

**Ranbaxy Research Laboratories, Gurgaon, India.**

Anticholinergics are the mainstay of the pharmacotherapy for the symptoms of chronic obstructive pulmonary disease (COPD). RBx 33B4088C a novel, M_3_-selective muscarinic receptor antagonist was evaluated *in vivo* for its efficacy, duration of action and possible synergism with PDE4 inhibitors subsequent to intratracheal instillation. RBx 33B4088C demonstrated potent bronchospasmolytic activity in rats, mice and guinea pigs with a duration of action >12 h in rats and >24 h in mice suggesting potential for once-daily dosing. Further, combination of an ineffective dose of RBx 33B4088C with an ineffective dose of RBx MNR 88281, a novel PDE4 inhibitor significantly reversed lipopolysaccharideinduced airway hyperreactivity. Thus, the promising preclinical pharmacological data suggests that RBx 33B4088C warrants further evaluation for its potential for treating the symptoms of COPD.

**690**

**Culture-independent specific detection of vancomycin resistant enterococci in surface waters by molecular beacon probe**

Lata P^1^, Ram S^1^, Agrawal M^2^, Shanker R^1^

**^1^Indian Institute of Toxicology Research, P. O. Box 80, Mahatma Gandhi Marg, Lucknow, U.P., ^2^Banaras Hindu University, Varanasi, U.P., India.**

Vancomycin-resistant-enterococci (VRE) are emerging environmental contaminants in the surface waters. The regulatory guidelines for recreational water quality recommend Enterococci as indicator of fecal contamination as their presence correlates best with the incidence of swimming-related gastroenteritis. The aim of this study was to develop a specific and rapid molecular beacon probe (MBP) based real-time polymerase chain reaction (PCR) assay for cultureindependent detection of VRE exhibiting vanA gene (encoding high level resistance against vancomycin) in environmental waters. The limit of detection (LOD) of the MBP assay was 20 CFU/mL [r = 0.980; PCR efficiency = 96.5%] in 2-fold dilution format with the minimal hands-on time of 2.5 hours. Specificity of the assay was demonstrated by efficient detection of environmental strains exhibiting VanA phenotype (n=25). An LOD enhancement technique involving water samples spiked with known concentration of reference strain DNA could improve the detection to 1 CFU/mL of VRE [r =0.943; PCR efficiency =99.7%]. VRE were detected from downstream surface waters of the rivers impacted by point sources of pollution and recreational activities. The probe detected VRE in root-mat associated microbiota of E. crassipes (Mart.) Solms, an aquatic nuisance weed, at eutrophic sites of the surface waters. In addition, the LOD enhancement technique enabled detection of otherwise non-detectable VRE concentration in the upstream sites of two Indian rivers. The assay developed can be used for sensitive and specific detection of VRE in environmental waters and identification of non-point sources of pollution for implementation of preventive measures to protect human health.

**691**

**Anti-inflammatory and analgesic activity of polyherbal formulation (madhumardan)**

Doshi Krunal Yogesh, ain Virendra K, Gulecha V S, Mahajan M S, Baheti J R

**SSDJ College of pharmacy, Neminagar, Chandwad, Nasik, India.**

The effect of PHF was investigated by perfprming various experiments of pain and inflammation. Anti-inflammatory activity of PHF (50, 100 and 300 mg/kg, p.o.) was studied in rats using carrageenan induced hind paw edema and formalin induced rat paw edema method. Analgesic activity of PHF (50,100 & 300 mg/ kg, p.o.) was studied in rats using acetic acid induced writhing, tail immersion method and hot plate method. PHF (50, 100, and 300 mg/kg) significantly (p<0.05) inhibited carrageenan induced hind paw edema and formalin induced rat paw edema. PHF (50, 100 & 300 mg/kg) significantly (p<0.05) reduced the number of writhing, increased latency to flick tail in tail immersion method and elevated the mean basal reaction time in hot plate method.

**692**

**Nootropic and antidepressant activity of polyherbal formulation**

Parikh V B, Gulecha V S, Mahajan M S, Gangurde H H, Randhir K, Baheti J R

**S. S. D. J College of Pharmacy, Neminagar Chandwad, Nasik, India.**

The effect of PHF was investigated in experimental models. Nootropic activity of PHF (50,100 and 300mg/kg, p.o.) was studied in mice using transfer latency in elevated plus maze (EPM), haloperidol induced catalepsy, lithium induced head twitches, object recognition method. Antidepressant activity of PHF (50, 100 and 300 mg/kg, p.o.) was studied in mice using forced swim method and haloperidol induced catalepsy method. PHF (50, 100, 300, mg/kg) significantly (*P*<0.05) indicate that polyherbal formultion, can facilitate learning and memory, and can be categorised as a nootropic agent. They also corroborate reduction in transfer latency in EPM, significant reduction in catalepsy score in haloperidol induced catalepsy; decrease in twitches in lithium induced head twitches method and PHF increase recognition memory in mice. PHF (50, 100, and 300mg/kg) significantly (*P*<0.05) shows decrease immobility time in forced swim method and significant reduction in catalepsy score in haloperidol induced catalepsy.

**693**

**Impact of teaching methodology on learning rational pharmacotherapy**

Patel T, Rege N, Kamat S, Shetty Y, Marathe P, Karve A, Patil A, Rokade R

**Seth G.S. Medical college & K.E.M. Hospital, Mumbai, India.**

**Objective:**

To study the impact on learning rational prescribing of a teaching methodology that utilizes case simulations and to compare it with traditional method.

**Methods:**

After obtaining Ethics Committee permission, 96 second MBBS participated in the study. Following a pre-test, students were divided into eight batches (12 students/batch) who were then exposed to case simulations. Each batch was taught four topics; two by using case simulations and two by traditional method in a random manner. A post test was held within one month of completion of the study for both exposed and non-exposed groups. The post-test included 3 problem cases, 2 related to the topics taught earlier and 1 to a new topic. Marks were assigned to the answers using a pre-determined model answer sheet.

**Results:**

The pre test did not show significant difference among the batches. In the post test, the marks obtained for objective questions in pharmacology were comparable for the students exposed to case simulations (n=96) and those exposed to traditional teaching (n=83). However the former group performed better (*P*<0.05) than the latter when they attempted a new problem (malaria). Among the 96 students, 48 were exposed to case simulations on Diabetes mellitus and peptic ulcer (Gp A) and the remaining by traditional method (Gp B). It was also observed that Gp B scored higher than Gp A(*P*< 0.05).

**Conclusion:**

The skill of rational prescribing is better acquired using case simulations than the traditional teaching. If practiced the skill can be extended to solve new therapeutic problems.

**694**

**Assessment of awareness of environmental mercury pollution among medical students at aiims, New Delhi**

Thejus T J, Gupta Y K

**All India Institute of Medical Sciences, New Delhi, India.**

Mercury is ubiquitous in environment and produces various toxic effects in human beings. However, in many developing countries like India most of the hospitals are still using mercury containing instruments. Hence the health care workers are exposed to mercury pollution through hospital wastes. A questionnaire based survey was done among medical students at All India Institute of Medical Sciences, New Delhi to assess the awareness about the proper handling and disposal of mercury containing items. All the questions were based on knowledge, use and disposal of mercury based products. The survey was conducted for three months and included 75 subjects. Students were selected randomly from the student register. The data collection was done by using the tool of pre-tested structured questionnaire containing 25 multiple choice questions. The collected data was coded and processed in the computer using Excel data sheet. Analysis was done using SPSS 12 soft ware. Majority can list out mercury related medical equipments but very low % is aware about the mercury content in vaccines and preservatives. 60% are aware that mercury can pollute air, water and soil. 96% agree that mercury is toxic to human being and 70% are having correct knowledge of route of mercury entry in the body. But only 33% is aware of mercury management policy. 84% had the knowledge Minamata disease. 85% answered the method for disposal of mercury waste as autoclaving. Medical and nursing undergraduates should be given better awareness about potential hazards of Hg and about how to manage mercury waste. It is also a public health concern.

**695**

**Antianxiety and antidepressant activity of polyherbal formulation**

Chordiya PV, Gulecha VS, Khandare RA, Gangurde HH, Baheti JR

**S. S. D. J College of Pharmacy, Neminagar Chandwad (Nasik), India.**

The effect of PHF was investigated in experimental models. Antianxiety activity of PHF (50,100 and 300mg/kg, p.o.) was studied in mice using open Field test, lithium induced head twitches, hole boared apparatus method. Antidepressant activity of PHF (50, 100 and 300 mg/kg, p.o.) was studied in mice using forced swim method and haloperidol induced catalepsy method. PHF (50,100, 300, mg/kg) significantly (*P*<0.05) indicate that polyherbal formultion, can facilitate learning and memory, and can be categorized as a Anxiolytic agent. The decrease in twitches in lithium induced head twitches method and PHF increase recognition memory in mice. PHF (50, 100, and 300mg/kg) significantly (*P*<0.05) shows decrease immobility time in forced swim method and significant increase in head pokeing in hole boared apparatus and square travels in open field apparatus.

